# Sequence of Reston Virus Isolate AZ-1435, an Ebolavirus Isolate Obtained during the 1989–1990 Reston Virus Epizootic in the United States

**DOI:** 10.1128/genomeA.01448-16

**Published:** 2017-01-12

**Authors:** Joseph P. Cornish, Larissa Diaz, Stacy M. Ricklefs, Kishore Kanakabandi, Jennifer Sword, Peter B. Jahrling, Jens H. Kuhn, Stephen F. Porcella, Reed F. Johnson

**Affiliations:** a Emerging Viral Pathogens Section, Division of Intramural Research, National Institute of Allergy and Infectious Diseases, National Institutes of Health, Fort Detrick, Frederick, Maryland, USA; b Genomics Unit, Research Technologies Section, National Institute of Allergy and Infectious Diseases, National Institutes of Health, Rocky Mountain Laboratories, Hamilton, Montana, USA; c Integrated Research Facility at Fort Detrick, National Institute of Allergy and Infectious Diseases, National Institutes of Health, Fort Detrick, Frederick, Maryland, USA

## Abstract

Reston virus (RESTV) was discovered in 1989–1990 during three connected epizootics of highly lethal viral hemorrhagic fever among captive macaques in primate housing facilities in the United States and Philippines. Currently, only one RESTV isolate from that outbreak (named Pennsylvania) has been sequenced. Here, we report the sequence of a second isolate, Reston virus/M.fascicularis-tc/USA/1990/Philippines89-AZ1435.

## GENOME ANNOUNCEMENT

Reston virus (RESTV; *Filoviridae*: *Ebolavirus*: *Reston ebolavirus* [1]) was discovered in 1989–1990, during a highly lethal viral hemorrhagic fever epizootic among crab-eating macaques (*Macaca fascicularis*) in primate-holding facilities in Reston, Virginia (1989 and 1990), Philadelphia, Pennsylvania (1989), and Alice, Texas (1989), USA. The affected animals were traced to a single primate export facility in the Philippines (2–5). RESTV reemerged in crab-eating macaques imported from the same facility in 1992 in Siena, Italy (6, 7), and in 1996 in Alice, Texas, USA (8–11), and was found in sick domestic pigs (*Sus scrofa*) and unspecified nonhuman primates in the Philippines in 2008 (12) and 2015 (13), respectively.

RESTV isolate sequence data are sparse (14), and only a single complete (15) genome (Reston virus/M.fascicularis-tc/USA/1989/Philippines89-Pennsylvania; RefSeq no. NC_004161 [16]) is available from the 1989–1990 epizootic. We sequenced RESTV AZ-1435, an isolate obtained from the spleen of a crab-eating macaque with signs of respiratory infection sampled during the 1990 Reston, Virginia, USA, episode of the 1989–1990 outbreak (17, 18). RESTV AZ-1435 was isolated by inoculating MA-104 cells with 10% spleen homogenate, followed by passage of MA-104 cell supernatant onto Vero E6 cells (17). We further passaged this sample by infecting Vero E6 cells and harvesting supernatant.

The virus was inactivated with Trizol LS (Thermo Fisher Scientific), and RNA was extracted as described previously (19). rRNA removal (low-input Ribo-Zero, Illumina) was performed prior to cDNA synthesis. cDNA libraries for sequencing were prepared using the Ovation RNA-Seq version 2 system (NuGEN Technologies) and the Ultralow DR (directional read) multiplex system (NuGEN Technologies) following the manufacturers’ recommendations. After single-primer isothermal amplification (SPIA, NuGEN Technologies), cDNA was purified using Agencourt RNAClean XP beads (Beckman Coulter, Inc. Life Sciences), sheared, and sequenced via paired-end (2 × 100 bp) in rapid chemistry mode on a HiSeq 2500 sequencing system (Illumina). SMARTer rapid amplification of cDNA ends (RACE) 5′/3′ kit (Clontech Laboratories, Inc.) was used to amplify the 5′ end of RNA. The 3′ end was amplified by adding adapter oligonucleotides with T4 RNA ligase I (New England BioLabs) and reverse transcriptase (RT)-PCR. RT-PCR products were cloned into pGEM T-easy vectors (Promega) for sequencing.

Trimmomatic (20), was used to trim reads for quality and length prior to assembly. Read quality was assessed pre- and post-trimming using open source FastQC version 0.11.5 software (Babraham Bioinformatics, http://www.bioinformatics.babraham.ac.uk/projects/fastqc). Reads were mapped to the RESTV reference genome (RefSeq no. NC_004161) using the Burrows–Wheeler aligner MEM (BWA-MEM) algorithm (21). Variants were identified using genome analysis toolkit (GATK) best practices (22, 23) and were manually inspected with Integrative Genomics Viewer (IGV) (24, 25). A final sequence was identified by comparison to NC_004161.

The RESTV AZ-1435 genome differs from the Pennsylvania genome at few locations, supporting previous investigations indicating that the 1989–1990 U.S. episodes were directly connected. Our data support the hypothesis that both RESTV isolates share an ancestor virus that was exported to all affected U.S. locations.

### Accession number(s).

The GenBank accession number of RESTV AZ-1435, now designated Reston virus/M.fascicularis-tc/USA/1989/Philippines89-AZ1435 based on current filovirus nomenclature standards (26), is KY008770.

## References

[1] Kuhn JH, Becker S, Ebihara H, Geisbert TW, Jahrling PB, Kawaoka Y, Netesov SV, Nichol ST, Peters CJ, Volchkov VE, Ksiazek TG 2011 Family *Filoviridae*, p 665–671. *In* King AMQ, Adams MJ, Carstens EB, Lefkowitz EJ (ed.), Virus taxonomy—ninth report of the International Committee on taxonomy of viruses. Elsevier/Academic Press, London, United Kingdom.

[2] Geisbert TW, Jahrling PB 1990 Use of immunoelectron microscopy to show Ebola virus during the 1989 United States epizootic. J Clin Pathol 43:813–816. doi:10.1136/jcp.43.10.813. 2229429 PMC502829

[3] Jahrling PB, Geisbert TW, Dalgard DW, Johnson ED, Ksiazek TG, Hall WC, Peters CJ 1990 Preliminary report: isolation of Ebola virus from monkeys imported to USA. Lancet 335:502–505. doi:10.1016/0140-6736(90)90737-P. 1968529

[4] Hayes CG, Burans JP, Ksiazek TG, Del Rosario RA, Miranda ME, Manaloto CR, Barrientos AB, Robles CG, Dayrit MM, Peters CJ 1992 Outbreak of fatal illness among captive macaques in Philippines caused by an Ebola-related filovirus. Am J Trop Med Hyg 46:664–671. 1621890 10.4269/ajtmh.1992.46.664

[5] Miranda ME, Miranda NL 2011 Reston ebolavirus in humans and animals in Philippines: a review. J Infect Dis 204(suppl 3):S757–S760. doi:10.1093/infdis/jir296. 21987747

[6] Ciorba A, Matteucci G, Perini L, Caristo ME, Brown D 1997 Infezione da virus Ebola nella scimmia: reperti clinici et anatomoistopatologici. Osservati nel corso del primo episidio verificatosi in Europa. Veterinaria (Cremona) 11:109–112.

[7] Perini L 2000 Outbreak of Ebola virus in Italy. Folia Primatol 71:280.

[8] Centers for Disease Control and Prevention 1996 Ebola-Reston virus infection among quarantined nonhuman primates—Texas, 1996. MMWR Morb Mortal Wkly Rep 45:314–316. 8602131

[9] Miranda ME, Ksiazek TG, Retuya TJ, Khan AS, Sanchez A, Fulhorst CF, Rollin PE, Calaor AB, Manalo DL, Roces MC, Dayrit MM, Peters CJ 1999 Epidemiology of Ebola (subtype Reston) virus in Philippines, 1996. J Infect Dis 179(suppl 1):S115–S119. doi:10.1086/514314. 9988174

[10] Miranda ME, Yoshikawa Y, Manalo DL, Calaor AB, Miranda NL, Cho F, Ikegami T, Ksiazek TG 2002 Chronological and spatial analysis of the 1996 Ebola Reston virus outbreak in a monkey breeding facility in Philippines. Exp Anim 51:173–179. doi:10.1538/expanim.51.173. 12012728

[11] Rollin PE, Williams RJ, Bressler DS, Pearson S, Cottingham M, Pucak G, Sanchez A, Trappier SG, Peters RL, Greer PW, Zaki S, Demarcus T, Hendricks K, Kelley M, Simpson D, Geisbert TW, Jahrling PB, Peters CJ, Ksiazek TG 1999 Ebola (subtype Reston) virus among quarantined nonhuman primates recently imported from the Philippines to the United States. J Infect Dis 179(suppl 1):S108–S114. doi:10.1086/514303. 9988173

[12] Barrette RW, Metwally SA, Rowland JM, Xu L, Zaki SR, Nichol ST, Rollin PE, Towner JS, Shieh WJ, Batten B, Sealy TK, Carrillo C, Moran KE, Bracht AJ, Mayr GA, Sirios-Cruz M, Catbagan DP, Lautner EA, Ksiazek TG, White WR, McIntosh MT 2009 Discovery of swine as a host for the Reston ebolavirus. Science 325:204–206. doi:10.1126/science.1172705. 19590002

[13] Geronimo JY 2015 Reston virus—Philippines (02): monkeys. ProMED-mail, archive no. 20150907.3629141 http://www.promedmail.org.

[14] Burk R, Bollinger L, Johnson JC, Wada J, Radoshitzky SR, Palacios G, Bavari S, Jahrling PB, Kuhn JH 2016 Neglected filoviruses. FEMS Microbiol Rev 40:494–519. doi:10.1093/femsre/fuw010. 27268907 PMC4931228

[15] Ladner JT, Kuhn JH, Palacios G 2016 Standard finishing categories for high-throughput sequencing of viral genomes. Rev Sci Tech 35:43–52. doi:10.20506/rst.35.1.2416. 27217167

[16] Kuhn JH, Andersen KG, Bào Y, Bavari S, Becker S, Bennett RS, Bergman NH, Blinkova O, Bradfute S, Brister JR, Bukreyev A, Chandran K, Chepurnov AA, Davey RA, Dietzgen RG, Doggett NA, Dolnik O, Dye JM, Enterlein S, Fenimore PW, Formenty P, Freiberg AN, Garry RF, Garza NL, Gire SK, Gonzalez JP, Griffiths A, Happi CT, Hensley LE, Herbert AS, Hevey MC, Hoenen T, Honko AN, Ignatyev GM, Jahrling PB, Johnson JC, Johnson KM, Kindrachuk J, Klenk HD, Kobinger G, Kochel TJ, Lackemeyer MG, Lackner DF, Leroy EM, Lever MS, Mühlberger E, Netesov SV, Olinger GG, Omilabu SA, Palacios G, Panchal RG, Park DJ, Patterson JL, Paweska JT, Peters CJ, Pettitt J, Pitt L, Radoshitzky SR, Ryabchikova EI, Saphire EO, Sabeti PC, Sealfon R, Shestopalov AM, Smither SJ, Sullivan NJ, Swanepoel R, Takada A, Towner JS, van der Groen G, Volchkov VE, Volchkova VA, Wahl-Jensen V, Warren TK, Warfield KL, Weidmann M, Nichol ST 2014 Filovirus RefSeq entries: evaluation and selection of filovirus type variants, type sequences, and names. Viruses 6:3663–3682. doi:10.3390/v6093663. 25256396 PMC4189044

[17] Jahrling PB, Geisbert TW, Jaax NK, Hanes MA, Ksiazek TG, Peters CJ 1996 Experimental infection of cynomolgus macaques with Ebola-Reston filoviruses from the 1989–1990 U.S. epizootic, p 115–134. *In* Schwarz TF, Siegl G (), Imported virus infections, vol. 11 Springer, Vienna, Austria. 10.1007/978-3-7091-7482-1_11 8800793

[18] Dalgard DW, Hardy RJ, Pearson SL, Pucak GJ, Quander RV, Zack PM, Peters CJ, Jahrling PB 1992 Combined simian hemorrhagic fever and Ebola virus infection in cynomolgus monkeys. Lab Anim Sci 42:152–157. 1318446

[19] Matsuno K, Weisend C, Travassos da Rosa APA, Anzick SL, Dahlstrom E, Porcella SF, Dorward DW, Yu XJ, Tesh RB, Ebihara H 2013 Characterization of the Bhanja serogroup viruses (*Bunyaviridae*): a novel species of the genus *phlebovirus* and its relationship with other emerging tick-borne phleboviruses. J Virol 87:3719–3728. doi:10.1128/JVI.02845-12. 23325688 PMC3624231

[20] Bolger AM, Lohse M, Usadel B 2014 Trimmomatic: a flexible trimmer for Illumina sequence data. Bioinformatics 30:2114–2120. doi:10.1093/bioinformatics/btu170. 24695404 PMC4103590

[21] Li H, Durbin R 2010 Fast and accurate long-read alignment with Burrows–Wheeler transform. Bioinformatics 26:589–595. doi:10.1093/bioinformatics/btp698. 20080505 PMC2828108

[22] DePristo MA, Banks E, Poplin R, Garimella KV, Maguire JR, Hartl C, Philippakis AA, del Angel G, Rivas MA, Hanna M, McKenna A, Fennell TJ, Kernytsky AM, Sivachenko AY, Cibulskis K, Gabriel SB, Altshuler D, Daly MJ 2011 A framework for variation discovery and genotyping using next-generation DNA sequencing data. Nat Genet 43:491–498. doi:10.1038/ng.806. 21478889 PMC3083463

[23] McKenna A, Hanna M, Banks E, Sivachenko A, Cibulskis K, Kernytsky A, Garimella K, Altshuler D, Gabriel S, Daly M, DePristo MA 2010 The genome analysis toolkit: a MapReduce framework for analyzing next-generation DNA sequencing data. Genome Res 20:1297–1303. doi:10.1101/gr.107524.110. 20644199 PMC2928508

[24] Robinson JT, Thorvaldsdóttir H, Winckler W, Guttman M, Lander ES, Getz G, Mesirov JP 2011 Integrative genomics viewer. Nat Biotechnol 29:24–26. doi:10.1038/nbt.1754. 21221095 PMC3346182

[25] Thorvaldsdóttir H, Robinson JT, Mesirov JP 2013 Integrative genomics viewer (IGV): high-performance genomics data visualization and exploration. Brief Bioinform 14:178–192. doi:10.1093/bib/bbs017. 22517427 PMC3603213

[26] Kuhn JH, Bao Y, Bavari S, Becker S, Bradfute S, Brister JR, Bukreyev AA, Chandran K, Davey RA, Dolnik O, Dye JM, Enterlein S, Hensley LE, Honko AN, Jahrling PB, Johnson KM, Kobinger G, Leroy EM, Lever MS, Mühlberger E, Netesov SV, Olinger GG, Palacios G, Patterson JL, Paweska JT, Pitt L, Radoshitzky SR, Saphire EO, Smither SJ, Swanepoel R, Towner JS, van der Groen G, Volchkov VE, Wahl-Jensen V, Warren TK, Weidmann M, Nichol ST 2013 Virus nomenclature below the species level: a standardized nomenclature for natural variants of viruses assigned to the family *Filoviridae*. Arch Virol 158:301–311. doi:10.1007/s00705-012-1454-0. 23001720 PMC3535543

